# Relationship between fasting glucose levels and in-hospital mortality in Chinese patients with acute myocardial infarction and diabetes mellitus: a retrospective cohort study

**DOI:** 10.1186/s12872-016-0331-2

**Published:** 2016-08-02

**Authors:** Hao Liang, Yi Chen Guo, Li Ming Chen, Min Li, Wei Zhong Han, Xu Zhang, Shi Liang Jiang

**Affiliations:** 1 The Ultrasonic Diagnosis and Treatment Department, Shandong Provincial Hospital affiliated to Shandong University, Jinan, Shandong China; 2 Department of Cardiology, Shandong Provincial Hospital affiliated to Shandong University, No.324, Jing Wu Wei Qi Road, Jinan, 250021 Shandong People’s Republic of China; 3 Department of Endocrinology, Shandong Provincial Hospital affiliated to Shandong University, Jinan, Shandong China

**Keywords:** Myocardial infarction, Diabetes mellitus, Glucose, Mortality

## Abstract

**Background:**

Previous studies have demonstrated that elevated admission and fasting glucose (FG) is associated with worse outcomes in patients with acute myocardial infarction (AMI). However, the quantitative relationship between FG levels and in-hospital mortality in patients with AMI remains unknown. The aim of the study is to assess the prevalence of elevated FG levels in hospitalized Chinese patients with AMI and diabetes mellitus and to determine the quantitative relationship between FG levels and the in-hospital mortality as well as the optimal level of FG in patients with AMI and diabetes mellitus.

**Methods:**

A retrospective study was carried out in 1856 consecutive patients admitted for AMI and diabetes mellitus from 2002 to 2013. Clinical variables of baseline characteristics, in-hospital management and in-hospital adverse outcomes were recorded and compared among patients with different FG levels.

**Results:**

Among all patients recruited, 993 patients (53.5 %) were found to have FG ≥100 mg/dL who exhibited a higher in-hospital mortality than those with FG < 100 mg/dL (*P* < 0.001). Although there was a high correlation between FG levels and in-hospital mortality in all patients (*r* = 0.830, *P* < 0.001), the relationship showed a J-curve configuration with an elevated mortality when FG was less than 80 mg/dL. Using multivariate logistic regression models, we identified that age, FG levels and Killip class of cardiac function were independent predictors of in-hospital mortality in AMI patients with diabetes mellitus.

**Conclusions:**

More than half of patients with AMI and diabetes mellitus have FG ≥100 mg/dL and the relationship between in-hospital mortality and FG level was a J-curve configuration. Both FG ≥ 100 mg/dL and FG <80 mg/dL were identified to be independent predictors of in-hospital mortality and thus the optimal FG level in AMI patients with diabetes mellitus appears to be 80–100 mg/dL.

## Background

Recent studies have shown that an elevated admission or fasting glucose (FG) level is common in patients with acute myocardial infarction (AMI) and is associated with an increased short-term mortality and a high incidence of congestive heart failure [1–5]. Among many clinical variables affecting short-term outcomes in patients with AMI, an elevated FG level, termed as stress hyperglycemia, has been identified as a new independent predictor of 30-day mortality in a group of patients with AMI and hyperglycemia [5]. This finding has important clinical significance because FG levels may serve as a simple marker to help clinicians stratify risk for optimal triage and management. Although hyperglycemia associated with AMI portends a gloomy short-term outcome in these patients, whether a low level of glucose would be a good omen for these subjects is unknown. Therefore, the quantitative relationship between FG levels across a wide range of values and the in-hospital mortality, and the optimal level of FG, in patients with AMI need to be clarified. The present study was carried out to assess the prevalence of elevated FG levels in hospitalized Chinese patients with AMI and diabetes mellitus and to determine the quantitative relationship between FG levels and the in-hospital mortality as well as the optimal level of FG in AMI patients with diabetes mellitus.

## Methods

### Patients

All patients who were admitted to our hospital between January 2002 and December 2013 with a definite diagnosis of AMI and diabetes mellitus and measurement of FG within 24 h of admission were included in the present study. The study was approved by the Medical Ethics Committee of Shandong Provincial Hospital Affiliated to Shandong University (NO.2015-034). No patients were directly involved in the study and no written informed consent was given by participants for their clinical records to be used in this study. All patients’ records/information was anonymized and de-identified prior to analysis. The diagnosis of AMI was based the following criteria [6]: Detection of a rise and/or fall of cardiac biomarker values (preferably cardiac troponin) with at least one value above the 99^th^ percentile upper reference limit and with at least one of the following: (1) Symptoms of ischaemia, (2) New or presumed new significant ST-segment–T wave changes or new left bundle branch block, (3) Development of pathological Q waves in the electrocardiogram, (4) Imaging evidence of new loss of viable myocardium or new regional wall motion abnormality, (5) Identification of an intracoronary thrombus by angiography or autopsy. Diabetes mellitus is diagnosed by demonstrating any one of the following [7]: (1) Fasting plasma glucose level ≥ 7.0 mmol/l (126 mg/dl), (2) Plasma glucose ≥ 11.1 mmol/l (200 mg/dl) two hours after a 75 g oral glucose load as in a glucose tolerance test, (3) Symptoms of hyperglycemia and casual plasma glucose ≥ 11.1 mmol/l (200 mg/dl). The exclusion criteria included development of AMI after percutaneous coronary intervention or coronary artery bypass grafting (CABG) or other cardiac operations.

### Data collection and definitions

A detailed review of the medical record for each patient was performed and clinical variables including baseline characteristics, in-hospital management, and incidence of adverse events during hospitalization were analyzed. Baseline characteristics consisted of age, gender, cigarette smoking, and history of angina pectoris, myocardial infarction, hypertension, family history of coronary artery disease, Killip class of cardiac function on admission, and FG and total cholesterol (TC) levels. The blood sample for FG and TC measurement was obtained after an overnight fast of ≥8 h within 24 h of admission and both FG and TC were determined enzymatically using an Auto Analyzer (Hitachi Inc). Patients were classified as normal FG group (FG <100 mg/dL) and elevated FG group (FG ≥100 mg/dL) according to the criteria of the 2003 follow-up report of the American Diabetes Association [8]. Analysis of in-hospital management involved application of reperfusion therapy with primary percutaneous coronary intervention or thrombolysis performed within 12 h of symptom onset, and medical therapy with the administration of β-receptor blockers, antiplatelets, heparins, angiotensin-converting-enzyme inhibitors (ACEI), angiotensin receptor blockers (ARB), statins and nitrates. Adverse outcomes of patients during hospitalization were defined as recurrent unstable angina pectoris, myocardial reinfarction, congestive heart failure and death. The diagnosis of unstable angina pectoris was established when patients had typical chest pain at rest with concomitant ischemic ST-T changes. Reinfarction was diagnosed based on the following criteria [6, 9]: (1) recurrent ST segment elevation or new pathognomonic Q waves appear, in at least two contiguous leads, particularly when associated with ischaemic symptoms for 20 min or longer, and (2) a second rise of CK-MB level to ≥2 times the upper normal limit, or an increase of >200 U/L of CK-MB level over the previous value if it had not dropped below the upper normal limit. Congestive heart failure was defined as Killip class of cardiac function ≥ II and a need for diuretic treatment at any time during hospitalization [10].

### Statistical analysis

Continuous variables were presented as means ± standard deviation (SD) and categorical variables as numbers and percentages. Comparisons between groups categorized by FG levels were made by 2-tailed Student *t* test for continuous data and by chi-square analysis for categorical variables. Spearman rank correlation analysis was applied to examine the quantitative relationship between FG levels and in-hospital mortality and multivariate logistic regression analysis used to identify risk factors that were predictive of in-hospital death independently. *P* value <0.05 was considered statistically significant. All analyses were performed with SPSS 13.0 (SPSS, Inc., Chicago, Illinois).

## Results

### Basic characteristics

A total of 1856 AMI patients with diabetes mellitus and measurement of FG within 24 h of admission were enrolled and there were 1301 men and 555 women aged from 25 to 91 years (63.2 ± 11.5 years). The basic characteristics in two groups of patients with normal and elevated FG were listed in Table 1. Compared with patients with normal FG, a greater proportion of patients with elevated FG were old and female, had a history of documented hypertension, and exhibited a high level of TC and Killip class of cardiac function. There was no significant difference in the history of angina pectoris and prior myocardial infarction, and family history of coronary artery disease between the two groups. In contrast, cigarette smoking was more common in patients with normal FG than those with elevated FG.

**Table 1:** Baseline characters in patients with fasting glucose <100 mg/dL and ≥ 100 mg/dL

Variables	Normal FG (*n* = 863)	Elevated FG (*n* = 993)	*P* value
Age (y)	61.7 ± 11.6	64.5 ± 11.2	<0.001
hospital stays (d)	9.6 ± 3.4	9.5 ± 3.4	0.677
TC(mg/dl)	192.5 ± 40.9	197.6 ± 46.6	0.013
Females	192 (22.2)	363(36.6)	<0.001
Hypertension	347(40.2)	452 (45.5)	0.021
Cigarette smoking	509 (59.0)	416 (41.9)	<0.001
Previous angina pectoris	551(63.8)	660(66.5)	0.237
Previous myocardial infarction	78(9.0)	95 (9.6)	0.696
Family history of CAD	208 (24.1)	205 (20.6)	0.074
Patients with Killip class ≥ III	46(5.3)	89 (9.0)	0.003

Data are mean values ± SD or number (%)

*FG* fasting glucose, *TC* total cholesterol, *CAD* coronary artery disease

### In-hospital management

The difference in in-hospital management between the two groups of patients was given in Table 2. Among 1856 patients with AMI and diabetes mellitus, only 708 underwent reperfusion therapy (38.1 %). It is noteworthy that patients with elevated FG received even less reperfusion therapy than those with normal FG. However, if the two reperfusion strategies were analyzed separately, there was no significant difference in receiving primary percutaneous coronary intervention, whereas the difference in receiving thrombolytic therapy was highly significant, between patients with normal and elevated FG. On the other hand, statins were used less frequently in patients with normal FG than those with elevated FG. There was no significant difference in the administration of other medications including antiplatelets, nitrates, β-receptor blockers, heparins, and ACEI or ARB between patients with normal and elevated FG.

**Table 2:** In-hospital management in patients with fasting glucose <100 mg/dL and ≥ 100 mg/dL

In-hospital Management	Normal FG (*n* = 863)	Elevated FG (*n* = 993)	*P* value
Reperfusion therapy	359 (41.6)	349 (35.1)	0.004
Thrombolysis	158 (18.3)	132 (13.3)	0.003
Primary PCI	201 (23.3)	217 (21.9)	0.46
Antiplatelets	847(98.1)	968 (97.5)	0.332
Nitrates	852 (98.7)	975 (98.2)	0.351
β-receptor blockers	610 (70.7)	664 (66.9)	0.077
ACEI or ARB	663 (76.8)	746 (75.1)	0.393
Statins	668 (77.4)	816 (82.2)	0.010
Heparins	787 (91.2)	900 (90.6)	0.676

*PCI* percutaneous coronary intervention, *ACEI* angiotensin-converting-enzyme inhibitor, *ARB* angiotensin receptor blocker, *GIK* glucose-insulin-potassium

### In-hospital adverse events

The incidence of adverse events in the two groups of patients during hospitalization was presented in Table 3. A higher incidence of in-hospital death (10.8 % vs 5.6 %, *P* < 0.001) and congestive heart failure (21.7 % vs 16.1 %, *P* = 0.002) was observed in patients with elevated FG than those with normal FG. In contrast, there was no significant difference in the incidence of recurrent unstable angina pectoris or reinfarction between the two groups.

**Table 3:** In-hospital Adverse Events in Patients with Fasting Glucose <100 mg/dL and ≥ 100 mg/dL

In-hospital adverse events	Normal FG (*n* = 863)	Elevated FG (*n* = 993)	*P* value
Unstable angina pectoris	341 (39.5)	363 (36.6)	0.190
Reinfarction	15(1.7)	24 (2.4)	0.309
Congestive heart failure	139 (16.1)	215 (21.7)	0.002
Total mortality	48 (5.6)	107 (10.8)	<0.001

**Fig. 1:**
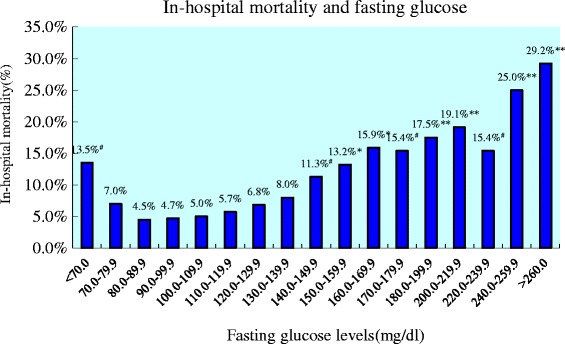
In-hospital mortality and fasting glucose. Figure 1 showed the relationship between FG levels and in-hospital mortality in all patients with AMI and diabetes mellitus. Chi-square analysis was used to test the difference in mortality between patients with a FG level of 80–89.9 mg/dL and any of the other patient groups with different FG levels. The in-hospital mortality was the lowest in patients with a FG level of 80–89.9 mg/dL and increased continuously with the increase in FG levels when FG was ≥100 mg/dL. However, the mortality tended to increase again when FG was <80 mg/dL. # *P* < 0.05, * *P* < 0.01, ** *P* < 0.001. FG: fasting glucose; AMI: acute myocardial infarction

**Fig. 2:**
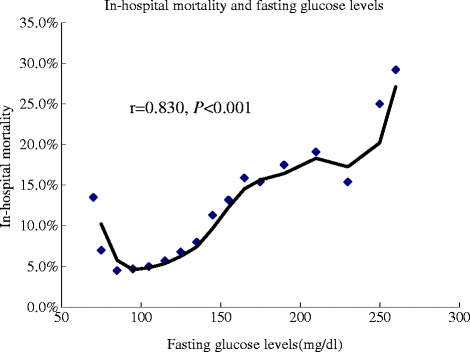
In-hospital mortality and fasting glucose levels. Spearman rank correlation between FG levels and in-hospital mortality in all patients with AMI and diabetes mellitus. There was a high correlation between the two parameters (*r* = 0.830, *P* < 0.001). However, the relationship showed a J-curve configuration and the mortality rose again when FG was <80 mg/dL. FG: fasting glucose; AMI: acute myocardial infarction

The in-hospital mortality among patients with different FG levels was 13.5 % (5/37 with FG <70 mg/dL), 7.0 % (14/199 with FG 70–79.9 mg/dL), 4.5 % (13/290 with FG 80–89.9 mg/dL), 4.7 % (16/337 with FG 90–99.9 mg/dL), 5.0 % (11/218 with FG100-109.9 mg/dL), 5.7 % (8/141 with FG 110–119.9 mg/dL), 6.8 % (8/118 with FG 120–129.9 mg/dL), 8.0 % (7/88 with FG 130–139.9 mg/dL), 11.3 % (9/80 with FG 140–149.9 mg/dL), 13.2 % (10/76 with FG 150–159.9 mg/dL), 15.9 % (7/44 with FG 160–169.9 mg/dL), 15.4 % (4/26 with FG 170–179.9 mg/dL), 17.5 % (10/57 with FG 180–199.9 mg/dL), 19.1 % (9/47 with FG 200–219.9 mg/dL), 15.4 % (4/26 with FG 220–239.9 mg/dL), 25.0 % (6/24 with FG 240–259.9 mg/dL), 29.2 % (14/48 with FG ≥260 mg/dL), respectively (Fig. 1). Spearman rank correlation analysis demonstrated a high positive correlation between FG levels and in-hospital mortality in all patients with AMI (*r* = 0.830, *P* < 0.001) (Fig. 2). However, the relationship between the two variables displayed a J-curve configuration and the lowest mortality occurred in patients with FG of 80–99.9 mg/dL. The in-hospital mortality showed a stepwise increase when FG was ≥ 100 mg/dL and tended to increase again when FG was < 80 mg/dL. Although there was no significant difference in mortality between patients with FG of 80–99.9 mg/dL and those with FG of 70–79.9 mg/dl (*P* = 0.182), there was significant difference in mortality between patients with FG of 80–99.9 mg/dL and those with FG <70 mg/dL (*P* = 0.046).

### Predictors of in-hospital mortality

In order to identify clinical variables that can independently predict the occurrence of in-hospital death in patients with AMI and diabetes mellitus, a multivariate logistic regression model was used in which in-hospital death was the dependent variable and risk factors including age, gender, history of hypertension, level of FG and TC, cigarette smoking, Killip class of cardiac function on admission, and administration of reperfusion therapy and statins, all of which showed a significant difference between patients with normal and elevated FG, were entered as independent variables. The results demonstrated that age, levels of FG and Killip class of cardiac function, history of hypertension, and administration of reperfusion therapy were significant predictors of in-hospital death in all patients with AMI and diabetes mellitus (Table 4). However, when multivariate logistic regression analysis was performed in patients with elevated FG, the prognostic value of history of hypertension disappeared and age, levels of FG, Killip class of cardiac function and administration of reperfusion therapy still were significant predictors (Table 5).

**Table 4:** Results of logistic regression analysis in all patients

Factors	OR (95 % CI)	*P* value
Age	1.10 (1.08–1.13)	<0.001
Gender	0.79 (0.50–1.25)	0.307
Fasting glucose	1.19 (1.12–1.26)	<0.001
TC	1.14 (0.93–1.39)	0.204
Cigarette smoking	1.50 (0.97–2.33)	0.068
Hypertension	1.83 (1.23–2.73)	0.003
Killip classification	3.48 (2.80–4.32)	<0.001
Reperfusion therapy	0.59 (0.42–0.83)	0.003
Statins	1.04 (0.53–2.02)	0.918

*OR* odds ratio, *CI* confidence interval

**Table 5:** Results of logistic regression analysis in patients with different fasting glucose levels

Factors	FG <80 mg/dL (*n* = 236)	FG 80–100 mg/dL (*n* = 627)	FG ≥ 100 mg/dL (*n* = 993)
	OR (95 % CI)	OR (95 % CI)	OR (95 % CI)
Age	1.08 (1.02–1.15)**	1.12 (1.06–1.18)**	1.11 (1.07–1.15)**
Gender	0.20 (0.02–1.82)^#^	076 (0.28–20.6)^#^	0.93 (0.52–1.67)^#^
Fasting glucose	0.13 (0.03–0.63)*	1.72 (0.46–6.50)^#^	1.25 (1.16–1.35)**
TC	1.08 (0.49–2.37)^#^	1.27 (0.80–2.00)^#^	1.11 (0.87–1.41)^#^
Cigarette smoking	2.22 (0.52–9.46)^#^	1.14 (0.46–2.81)^#^	1.56 (0.89–2.75)^#^
Hypertension	2.89 (0.88–9.51)#	2.47 (1.07–5.74)*	1.54 (0.92–2.57)^#^
Killip classification	4.93 (2.35–10.33)**	2.21 (1.37–3.56)**	4.06 (3.05–5.40)**
Reperfusion therapy	0.54 (0.20–1.48)^#^	0.66 (0.35–1.26)^#^	0.61 (0.38–0.98)*
Statins	1.11 (0.19–6.49)^#^	1.23 (0.30–4.94)^#^	1.08 (0.44–2.67)^#^

#*P* > 0.05, *P < 0.05, **P < 0.01

To further explore the prognostic value of a low level of FG, we performed multivariate logistic regression analysis in the subgroup of patients with FG <80 mg/dL. There were 236 patients in this subgroup with 193 men and 43 women aged 61.1 ± 11.7 years. The dependent and independent variables were the same as previous analysis. The result demonstrated that only age, FG levels and Killip class of cardiac function were identified as the independent predictors of in-hospital mortality in this subgroup of patients (Table 5). However, the level of FG lost its prognostic value in the subgroup of patients with normal FG (Table 5). As we mentioned above, age and Killip class of cardiac function on admission, FG levels were significant predictors of in-hospital mortality in patients with AMI and diabetes mellitus regardless of the FG level was low or high.

Hemoglobin A1c (HbA1c), an indirect measure of mean blood glucose over the previous 2–3 months, does not require fasting, and is more reproducible than FG and the American Diabetes Association (ADA) report in 2009 advocated the diagnosis of diabetes may be based on A1c ≥ 6.5 %. When we reviewed the medical record for each AMI patient we found that HbA1c was measured 439 patients and there were 332 men and 107 women aged from 27 to 91 years (63.1 ± 12.1 years). The mean HbA1c level of the study population was 5.73 ± 1.42 % (range: 3.81–11.35 %) and HbA1c level < 6.5 % was found in 328 patients. Compared with patients with HbA1c level < 6.5 %, a greater proportion of patients with HbA1c level ≥ 6.5 % were old (64.9 ± 12.4 years vs 62.5 ± 11.9 years, *P* = 0.067) and female (39.6 % vs 19.2 %, *P* < 0.001), and exhibited a high level of FG (167.3 ± 47.4 mg/dL vs 92.0 ± 16.6 mg/dL, *P* < 0.001) and TC (223.0 ± 48.8 mg/dL vs 210.0 ± 38.5 mg/dL, *P* = 0.004). In contrast, cigarette smoking was more common in patients with HbA1c level < 6.5 % than those with HbA1c level ≥ 6.5 % (60.7 % vs 45.0 %, *P* = 0.004). Among this study population, only 181 underwent reperfusion therapy (41.2 %). It is noteworthy that patients with HbA1c level ≥ 6.5 % received even less reperfusion therapy than those with HbA1c level < 6.5 % (26.1 % vs 46.3 %, *P* < 0.001). There was no significant difference in receiving other life-saving therapies between the two groups of patients. A higher incidence of in-hospital death was observed in patients with HbA1c level ≥ 6.5 % than those with HbA1c level < 6.5 % (18.0 % vs 7.0 %, *P* < 0.001). In contrast, there was no significant difference in the incidence of congestive heart failure (17.1 % vs 13.7 %, *P* = 0.381), recurrent unstable angina pectoris (36.0 % vs 43.0 %, *P* = 0.198) and reinfarction (4.5 % vs 2.1 %, *P* = 0.324) between the two groups. Using multivariate logistic regression models, we identified that age, Killip class of cardiac function and reperfusion therapy were independent predictors of in-hospital mortality in this study population, fasting glucose concentration and HbA1c level were not independent predictors.

Up till now, only 835 patients have completed the follow-up which included 322 patients in the normal FG group and 513 patients in the elevated FG group. A higher incidence of death was observed in patients with elevated FG than those with normal FG during the long term follow-up (32.4 % vs 25.2 %, *P* = 0.026). Logistic regression showed that Killip class of cardiac function, age, gender, and fasting glucose levels were independent predictors of long term survival in the follow-up population. Only 82 patients have completed the follow-up in the subgroup of patients with FG <80 mg/dL and logistic regression analysis demonstrated that FG levels was not independent predictor of long term mortality in the subgroup of patients.

## Discussion

Since the occurrence of fasting hyperglycemia is associated with an untoward outcome in patients with AMI, accurate detection of this co-morbidity is of great importance for optimal triage and management. The major finding in the present study was that more than half of the Chinese patients with AMI and diabetes mellitus had an elevated FG level which was associated with higher in-hospital mortality than in those with a normal FG level. Although there was a high correlation between FG levels and in-hospital mortality in all patients, the relationship was a J-curve configuration with an increased mortality when FG was <80 mg/dL. Both FG ≥ 100 mg/dL and FG <80 mg/dL were identified to be independent predictors of in-hospitality and thus the optimal FG level in patients with AMI appears to be 80–100 mg/dL. To the best of our knowledge, this study is the first in the literature to report the J-curve relationship between FG levels and in-hospital mortality and define the optimal FG levels in Chinese patients with AMI and diabetes mellitus.

In the present study, a higher incidence of in-hospital death and congestive heart failure was found in AMI patients with elevated FG than those with normal FG. Although differences in baseline characteristics and management may contribute to the higher mortality in patients with elevated FG, the level of FG as a continuous variable remained a strong independent predictor of in-hospital death in this group of patients even after all risk factors were taken into account in our multivariate logistic regression model. It is noteworthy that the in-hospital mortality increased continuously with the increase in FG across a wide range of FG measurements and there was a high correlation between FG levels and in-hospital mortality in all patients. It should also noted that the level of FG lost its prognostic value in patients with normal FG and the stepwise increase in mortality was not statistically significant until FG reached a level of ≥ 140 mg/dL (Fig. 1). These results suggest that FG <100 mg/dL may not be a risk factor of in-hospital death in patients with AMI, a finding similar to previous studies [5, 11]. However, the relationship between FG levels and in-hospital mortality revealed a J-curve configuration and the mortality tended to increase again when FG was < 80 mg/dL. Multivariate logistic regression analysis in this subgroup of patients demonstrated that FG < 80 mg/dL was also an independent predictor of in-hospital mortality in patients with AMI. This new finding indicates that a low albeit normal FG level may do harm to patients with AMI. Since both FG ≥ 100 mg/dL and FG <80 mg/dL were independent predictors of in-hospitality in patients with AMI and diabetes mellitus, it is logical to speculate that the optimal FG level in patients with AMI and diabetes mellitus should be 80–100 mg/dL.

The mechanisms underlying the relationship between FG levels and in-hospital mortality in patients with AMI and diabetes mellitus are not completely understood. It has been suggested that hyperglycemia is a marker of extensive myocardial damage and a consequence of excessive secretion of stress hormones that inhibit the action of insulin [12]. Alternatively, hyperglycemia may result from insulin resistance commonly seen in patients with coronary artery disease, which is aggravated by the onset of AMI. Insulin deficiency promotes lipolysis and increases circulating free fatty acids which may be toxic to ischemic myocardium. Recent studies have showed that insulin has a powerful anti-inflammatory effect, which is associated with improvements in morbidity and mortality [13], and these beneficial effects may have been reduced in patients with elevated FG. Moreover, fasting hyperglycemia per se may induce dysfunction of vascular endothelial cells, vascular smooth muscle cells and platelets [14], and cause hypercoagulability and impaired fibrinolysis [15]. The reason why a low-normal FG level carries a poor outcome in patients with AMI is not clear. The relatively low plasma glucose levels in these patients are probably be attributed to the administration of antidiabetic agents, insufficient food intake and/or excessive vomiting. When we reviewed the medical record we found the excessive administration of antidiabetic agents, insufficient food intake and/or excessive vomiting were seen in about one fourth of patients having low blood glucose levels. A low level of plasma glucose may reduce the glucose intake by the ischemic myocardium and impair remote ischemic preconditioning and further exacerbate myocardial damage [16].

Among 22 clinical parameters recorded in this study, we identified age, levels of FG and Killip class of cardiac function, history of hypertension, and administration of reperfusion therapy as significant predictors of in-hospital death in patients with AMI and diabetes mellitus. As mentioned in this study, compared with patients having lower FG levels, a greater proportion of patients with elevated FG were old and female, had a history of documented hypertension. Hemorrhage, especially cerebral hemorrhage was more common in old, female, hypertensive patients when thrombolytic therapy was administered. Thus patients with elevated FG received even less thrombolytic therapy than those having lower FG levels.

The odds ratio of FG was 1.19 in patients with elevated FG which is not as impressive as in the previous studies because FG levels were entered as the continuous variables in our multivariate logistic regression model whereas they were entered as categorical variables in previous studies [5]. However, as judged by their corresponding *P* values, the predictive power of FG levels was weaker than age and Killip class of cardiac function but stronger than the administration of reperfusion therapy and history of hypertension.

It is well known that HbA1c captures chronic hyperglycemia in the prior 2–3 months, is well correlated to chronic diabetes complications, and has less preanalytical problems and biological variability than plasma glucose, with a noninferior standardization. As shown in this study, high level of HbA1c is associated with an increased short-term mortality in patients with AMI and diabetes mellitus. However, multivariate logistic regression analysis demonstrated that HbA1c level was not an independent predictor of in-hospital mortality in the subgroup of patients whose HbA1c level and FG were all measured during hospital stay. The reason for this may be due the small sample size studied in this subgroup of patients because in contrast to seen in the whole population, like HbA1c level, FG was not an independent predictor in this subgroup of patients too.

Although most of patients lost to follow-up and we keep in touch with only 835 patients till now, high incidence of death was observed in patients with elevated FG during the long term follow-up and logistic regression showed that FG was also an independent predictor of long term survival in this subgroup population. This result showed that FG level was not only a short term but also a long term independent predictor of outcomes in patients with AMI and diabetes. This finding seems to be particularly important due to a limited number of proven predictors of mortality in these patients.

### Study limitations

There were several important limitations in our study. First, this is a retrospective and observational study. However, all the medical records were made by the same department of cardiology and laboratory of biochemistry and the quality control can thus be assured. Besides, our study provides unbiased data in real clinical practice. Second, the majority (61.9 %) of patients enrolled did not undergo reperfusion therapy because of late arrival at the hospital or economical restraint, which reflects the therapeutic situations in the past 12 years in Shandong Province of China. However, the in-hospital mortality in our patients was comparable to previous reports. Third, although all patients hospitalized between January 2002 and December 2013 were enrolled, patients who died before reaching the hospital may have been missed, which may have resulted in an underestimation of the mortality rate in patients with AMI and diabetes mellitus.

## Conclusions

In conclusion, more than half of the Chinese patients with AMI and diabetes mellitus had elevated FG and there was higher in-hospital mortality in these patients than in those with AMI and a normal FG level. The in-hospital mortality in patients with AMI and diabetes mellitus increases continuously with the increase in FG and there was a high correlation between the two parameters. However, the relationship was a J-curve configuration with an increased mortality when FG was FG <80 mg/dL. Both FG ≥ 100 mg/dL and FG <80 mg/dL were identified to be independent predictors of in-hospitality and thus the optimal FG level in patients with AMI and diabetes mellitus appears to be 80–100 mg/dL. Long term follow-up study demonstrated that FG level was also a long term independent predictor of outcomes in patients with AMI and diabetes.

## Abbreviations

ACEI, angiotensin-converting-enzyme inhibitors; AMI, acute myocardial infarction; ARB, angiotensin receptor blockers; CABG, coronary artery bypass grafting; CAD, coronary artery disease; CI, Confidence intervals; FG, fasting glucose; GIK, Glucose-Insulin-Potassium; OR, odds ratio; PCI, percutaneous coronary intervention; SD, standard deviation; TC, total cholesterol
